# Perforation of Meckel’s diverticulum by a fish bone presenting as acute appendicitis: a case report

**DOI:** 10.1186/1752-1947-7-231

**Published:** 2013-10-02

**Authors:** Ioannis Dimitriou, Neofytos Evaggelou, Elefteria Tavaki, Eftymios Chatzitheoklytos

**Affiliations:** 1 Department of General Surgery, General Hospital of Thessaloniki, O Agios Dimitrios Kanari 7 Stavroupoli, Thessaloniki PC56430, Greece

**Keywords:** Meckel’s diverticulum, Perforation, Acute appendicitis

## Abstract

**Introduction:**

Meckel’s diverticulum is the commonest congenital abnormality of the gastrointestinal tract. Most of them are asymptomatic but can rarely present with forms of complications such as bleeding, obstruction, diverticulitis, intussusception and neoplasm. Patients with a perforation of Meckel’s diverticulum by a foreign body are rare and may present with right iliac fossa pain, which mimics acute appendicitis.

**Case presentation:**

A 64-year-old Greek man presented with an eight-hour history of right iliac fossa pain. On examination, our patient had tenderness in his right iliac fossa. A provisional diagnosis of acute appendicitis was made. He was taken to theatre with the option of an appendicectomy. His appendix was found to have an about normal appearance. An inflamed Meckel’s diverticulum that had been perforated by a fish bone was found to be the cause of the abdominal pain. A Meckel’s diverticulectomy was performed. Our patient made an uneventful recovery and was discharged after two days.

**Conclusions:**

Complications of Meckel’s diverticulum can be difficult to diagnose and early recognition and timely operative intervention must occur in order to provide the best outcome for these patients. This is an interesting and unusual case of perforation of Meckel’s diverticulum that highlights the importance of considering Meckel’s diverticulum as a differential diagnosis in every patient presenting with acute abdomen.

## Introduction

Meckel’s diverticulum is the most prevalent congenital anomaly of the gastrointestinal tract, affecting approximately 2% of the general population. A 3:2 male to female ratio has been reported. Meckel’s diverticula are designated true diverticula because their walls contain all of the layers found in normal intestine. Their location varies among individual patients, but they are usually found in the ileum within 100cm of the ileocecal valve. Approximately 60% of Meckel’s diverticula contain heterotopic mucosa, of which over 60% consist of gastric mucosa [1]. Other heterotopic tissues include pancreatic tissue, which is found in 6% of cases, jejunal, duodenal mucosa or Brunner’s glands, with every one of this type found in 2% of cases, and pancreatic islets, colonic mucosa, endometriosis and hepatobiliary tissue, which are found in smaller percentages [2]. A commonly quoted ‘rule of twos’ also applies: 2% of the population have the anomaly, it is approximately 2 inches in length, it is usually found within 2 feet proximal to the ileocecal valve, it is often found in children under two years of age, it contains two types of common ectopic tissue (gastric and pancreatic) and it affects males twice as often as females [3].

The overall lifetime complication rate is approximately 4% [4]. The most common presentation associated with symptomatic Meckel’s diverticula is bleeding, followed by intestinal obstruction, diverticulitis, intussusceptions and neoplasm [1-3]. However, perforation is very rarely seen and, in a review, was reported as being responsible for 0.5% of symptomatic diverticula [2]. This is an interesting and unusual case of perforation of Meckel’s diverticulum mimicking acute appendicitis, a very rare complication caused by a fish bone. To the best of the authors’ knowledge, this is the first reported case of perforation of Meckel’s diverticulum by a fish bone in Greece and from an extensive review of the literature, such an unusual complication of Meckel’s diverticulum has rarely been reported [2,5].

## Case presentation

A 64-year-old Greek man presented with an eight-hour history of right iliac fossa pain. He denied any other symptoms apart from anorexia and nausea over the last six hours. His only significant past medical history was that of a laparoscopic cholocystectomy five years ago, and he was taking Lipemia syrup for hyperlipidemia.

On his admission, his vital signs were within normal range. A physical examination demonstrated tenderness in the right iliac fossa, rebound tenderness and a positive McBurney’s sign. His blood analysis revealed an elevated blood count, his white blood cells (WBC) were 14,440/μl (normal values 4.6 to 10.2 × 10^3^/mL) and 74% of them were neutrophiles (normal values 40 to 75%). The rest of the routine preoperative blood tests and his erect chest and abdominal X-rays were unremarkable. A provisional diagnosis of acute appendicitis was made and initial management included intravenous fluid resuscitation and antibiotic coverage. No other examinations were performed and, after our patient gave his written consent, he was taken to the operating theatre for an open appendectomy under general anaesthesia.

**Figure 1:**
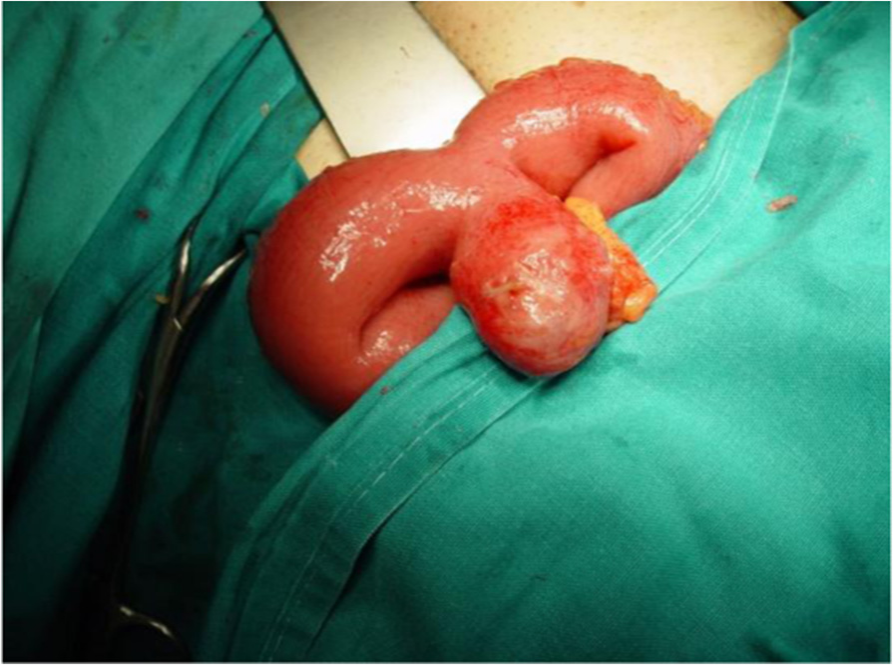
Meckel’s diverticulum perforated by a fish bone as it was found during surgery.

**Figure 2:**
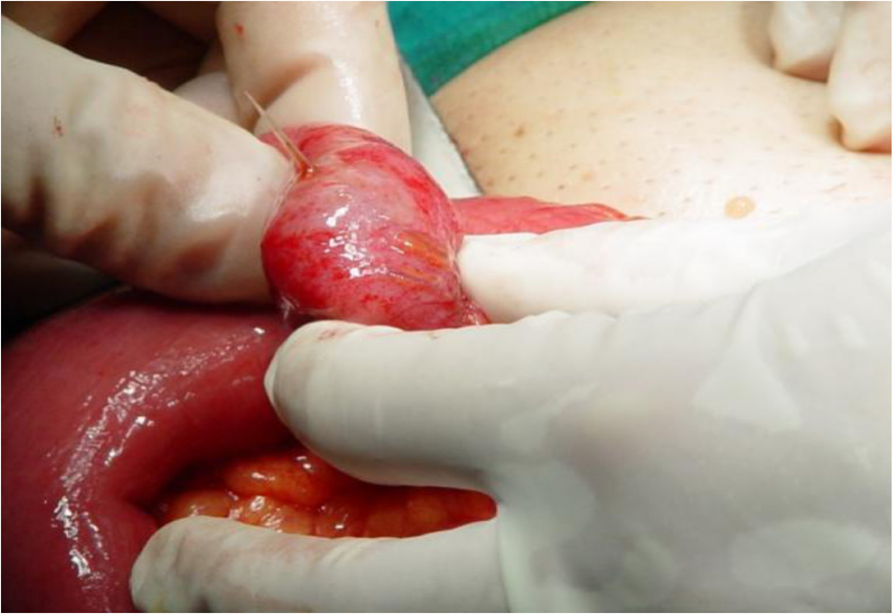
A view of the fish bone that perforated the Meckel’s diverticulum.

**Figure 3:**
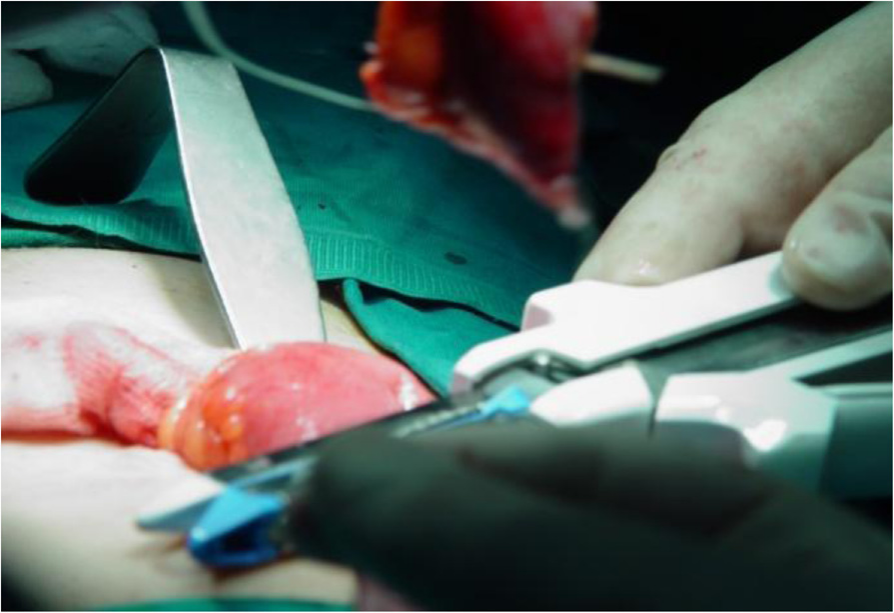
Excising the Meckel’s diverticulum using a GIA 55mm.

**Figure 4:**
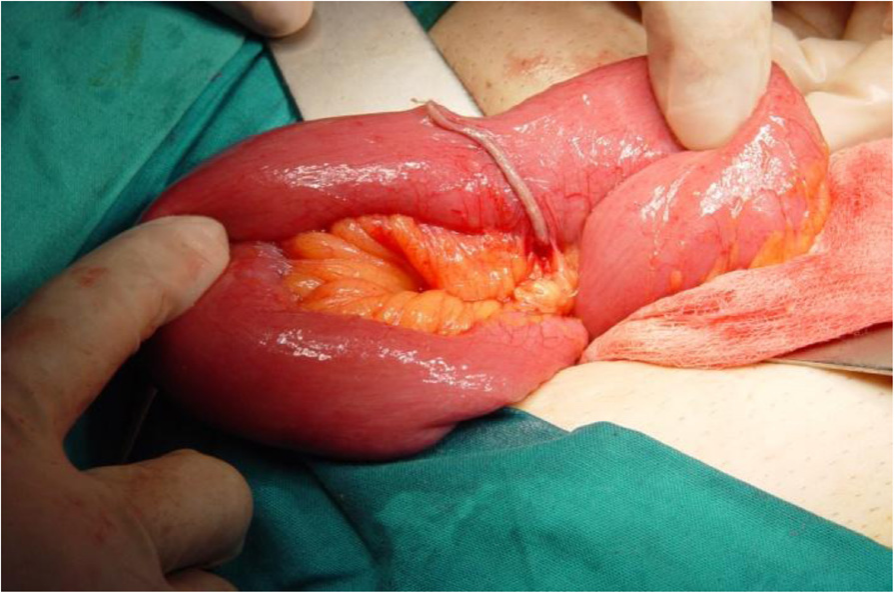
The ileum after the excision of the Meckel’s diverticulum.

A McBurney incision was performed and a normal-appearing appendix was identified, which did not have any remarkable sign of inflammation that could explain the tenderness and the peritoneal irritation. During the operation, some serous peritoneal fluid was observed between the small intestine loops. A typical appendectomy was performed. An examination of the small bowel revealed an inflamed and perforated Meckel’s diverticulum due to a foreign body approximately 100cm proximal to the ileocecal valve (Figure 1). The Meckel’s diverticulum had been perforated by a foreign body, which was found to be a fish bone (Figure 2). A Meckel’s diverticulectomy was performed using a gastrointestinal anastomosis stapler (GIA 55mm) (Figures 3 and 4). The operational site was irrigated with normal saline solution.

**Figure 5:**
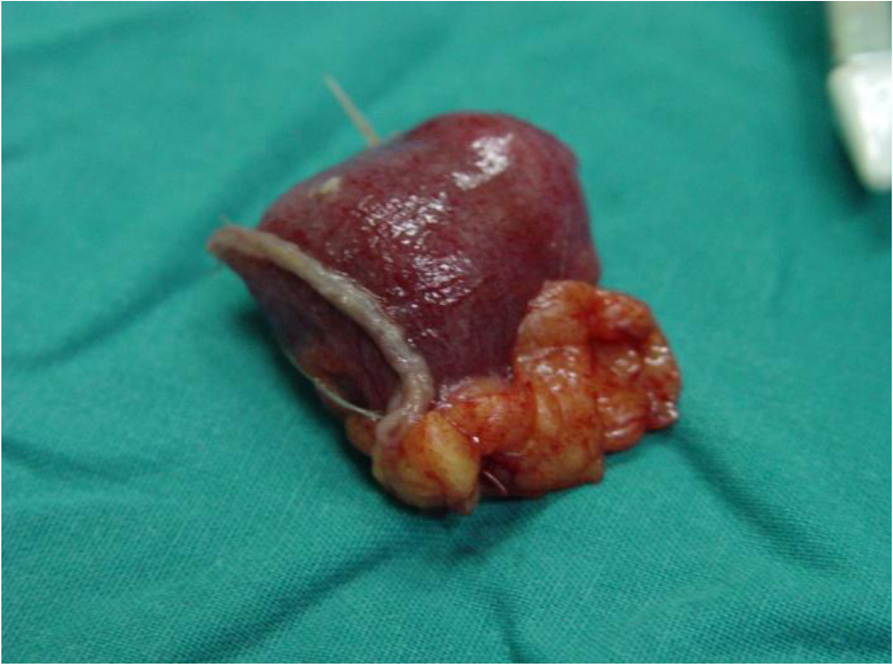
The anatomical specimen after excision.

The sectioned specimen (Figure 5) showed no ectopic tissue in the diverticulum wall. The patient made an uneventful recovery postoperatively and was discharged on the second postoperative day. At the follow-up examination 10 days and 1 month after surgery he was doing well.

## Discussion

Meckel’s diverticulum is a congenital, intestinal blind pouch that results from an incomplete obliteration of the vitelline duct during the fifth week of gestation. Wilhelm Fabricius Hildanus, a German surgeon, first described the diverticulum in 1598. However, the entity was not named until 1809, when Johann Friedrich Meckel the Younger first reported his research on the diverticulum’s anatomy and embryology. Furthermore, Meckel showed that incomplete obliteration of the vitelline duct results in not only Meckel’s diverticulum but also enterocysts, intestinal-umbilical fistulas, and mesodiverticular bands [6]. Although it generally remains silent, life-threatening complications may arise, making it important to have a detailed knowledge of its anatomical and pathophysiological structure and properties to deal with such complications [7].

Bleeding is the most common presentation in children, representing 50% of Meckel’s diverticulum-related complications among patients younger than 18 years of age. Intestinal obstruction is the most common presentation in adults with Meckel’s diverticula. Diverticulitis, present in 20% of patients with symptomatic Meckel’s diverticula, is associated with a clinical syndrome that is indistinguishable from acute appendicitis [1,3].

Meckel’s diverticulum is notoriously difficult to diagnose both clinically and radiologically as the symptoms and imaging features are non-specific. The utility of Tc-99m pertechnetate scintigraphy in the diagnosis of ectopic gastric mucosa is well established, particularly in the case of Meckel’s diverticulum, despite substantial variation in the reported sensitivity. Radiological as well as clinical findings aid clinicians in diagnosing this congenital anomaly [5].

A very small percentage of ingested foreign bodies can cause perforation of the bowel, leading to acute abdomen requiring surgical intervention. Foreign bodies such as dentures, fish bones, chicken bones, toothpicks and cocktail sticks have been known to cause bowel perforation. There are more than 300 cases in the literature of bowel perforation caused by foreign bodies. The majority of patients do not recall ingesting the foreign body, and it is discovered either on investigation (abdominal X-ray or computed tomography [CT] scan), or during an operation [8].

The most common site of perforation is the terminal ileum and colon, although an increased incidence of perforation has been reported in association with Meckel’s diverticulum, the appendix and diverticular disease. Perforation of Meckel’s diverticulum is usually caused by foreign bodies, a very rare complication as most foreign bodies pass through the gastrointestinal tract without any consequences. Perforation of Meckel’s diverticulum caused by a fish bone has rarely been reported in the literature [9,10].

Perforation of Meckel’s diverticulum by foreign bodies is extremely rare and in a review, the indication rate for a resection due to perforation by a foreign body was reported to be 8% of all complicated diverticula [11]. There are several case reports in the literature regarding perforations caused by toothpicks, fish bones, chicken bones, needles, pins, wood splinters, seeds, parasites, tomato skin, a button battery, and an alkaline battery [11-13]. There seems to be a tendency for foreign bodies to lodge in the blind pouch of Meckel’s diverticulum [5]. The mechanism of perforation, as it was suggested by Ward-McGuard, is a combination of local inflammation due to irritation of the foreign body, and pressure necrosis of the divertculum wall, secondary to attempts by peristalsis to push the foreign body toward the tip of the diverticulum [14].

Perforation of Meckel’s diverticulum remains a differential diagnosis of right iliac fossa pain. Perforation of Meckel’s diverticulum caused by a fish bone is a very rare complication and may lead to a fatal outcome if not recognised early [5]. In all previously reported cases in the literature, the diagnosis of foreign body perforation of Meckel’s diverticulum was only made when patients underwent surgery for suspected acute appendicitis. We therefore report this rare case in the hope that it may remind the physician of this interesting anomaly when evaluating the acute abdomen by preoperative CT scan [2].

## Conclusions

Complications of Meckel’s diverticulum are uncommon and can be difficult to diagnose. Early recognition and timely operative intervention must occur in order to provide the best outcome for these patients. Meckel’s diverticulum perforation by a foreign body is a very rare condition; however, it should be kept in mind as a differential diagnosis for every patient presenting with acute abdomen, especially those with symptoms mimicking acute appendicitis or bowel perforation.

## Consent

Written informed consent was obtained from the patient for publication of this case report and any accompanying images. A copy of the written consent is available for review by the Editor-in-Chief of this journal.

## Competing interests

The authors declare that they have no competing interests.

## Authors’ contributions

ID wrote the entire case report, performed the literature review, drafted the review, took intraoperative photos, participated in the operation as third surgeon (first assistant) and was in charge of the patient. NE assisted in writing the case, assisted with the literature search, drafted the review, obtained the consent of the patient and performed the operation as second surgeon. ET performed the operation as primary surgeon and assisted in processing the digital images. EC provided overall supervision, direction and suggestions for the case report, and is the unit consultant who had overall responsibility for the care of the patient. All authors read and approved the final manuscript.
